# *Vibrio alginolyticus* is the pathogen of “Baotou” disease causing serious damage to *Gracilariopsis lemaneiformis* cultivation in China

**DOI:** 10.1128/mbio.03120-24

**Published:** 2024-12-11

**Authors:** Tong Pang, Feng Wang, Qunqun Guo, Mengjie Zhang, Yuanyuan Sun, Jianguo Liu

**Affiliations:** 1 Key Lab of Breeding Biotechnology and Sustainable Aquaculture (CAS), CAS and Shandong Key Laboratory of Experimental Marine Biology, Institute of Oceanology, Chinese Academy of Sciences, Qingdao, China; 2 Laboratory for Marine Biology and Biotechnology, Qingdao Marine Science and Technology Center, Qingdao, China; 3 College of Life Sciences, Qingdao University 12593 , Qingdao, China; University of Nebraska-Lincoln, Lincoln, Nebraska, USA

**Keywords:** *Gracilariopsis lemaneiformis*, *Vibrio alginolyticus*, "Baotou" disease, pathogen

## Abstract

In recent years, a disease called “Baotou” has been causing large-scale yield reductions of *Gracilariopsis lemaneiformis* in China. Interestingly, *Vibrio alginolyticus*, which once reported to be a probiotic or pathogen for multiple marine organisms, was strongly proved to be the pathogen causing “Baotou” disease in this study. Analysis of 16S rRNA gene profiling revealed that *V. alginolyticus* was the most abundant and dominant bacterium on the algal thalli suffering from “Baotou” disease, whereas its presence was scarcely detected on healthy thalli. Scanning electron microscope (SEM) analysis revealed that a large number of *V. alginolyticus* cells were found to be attached to the algal thalli with “Baotou” disease and the rotten thalli acquired by the lab infection treatment. *V. alginolyticus* could cause the rotten symptoms which were consistent with those of “Baotou” disease. According to Koch’s postulates, *V. alginolyticus* was identified as the pathogen causing “Baotou” disease.

**IMPORTANCE:**

A highly contagious disease known as “Baotou” disease has persistently triggered significant yield reductions in *G. lemaneiformis* throughout China. The pathogen of “Baotou” disease was isolated and identified as *V. alginolyticus* in this study. Interestingly, *V. alginolyticus* was once reported to be a probiotic to *Saccharina japonica*, and pathogen to *Haliotis diversicolor*, *Paleopneustes cristatus*, *Ruditapes decussatus,* and *Litopenaeus vannamei*. The study indicates that *V. alginolyticus* play a significant role in the competition or co-existence between *G. lemaneiformis*, *S. japonica*, abalone, sea urchin, bivalve, and shrimp.

## INTRODUCTION

Seaweeds can grow rapidly in the offshore farm without occupying land and are rich in various chemical compositions that are useful to humans (1, 2). The productivity of seaweed increased significantly from 10.6 million tons in 2000 to 35 million tons in 2020, dominated especially by *Saccharina japonica*, *Eucheumatoid* species, and *Gracilarioids* species (3). The *Gracilariopsis lemaneiformis*, a species of *Gracilarioids* algae, is widely distributed in coastal countries all over the world such as the China, America (4, 5). It not only serves as a raw material for agar production and feed for abalone aquaculture but also plays a crucial role in regulating and restoring the ecological environment, thus bearing significant importance for human production and livelihoods (6, 7). The total area of cultivated *G. lemaneiformis* in China exceeded 13,000 hectares in 2022 (8). *G. lemaneiformis* has become the second largest main seaweed species planted after kelp species in China (9).

In recent years, with the expansion of *G. lemaneiformis* breeding industry, various algal rot diseases have been frequently and continuously affecting the cultivation of *G. lemaneiformis* (10–12). It is noticed that a rot disease named “Baotou” has been affecting the production of Chinese *G. lemaneiformis* since 2015 (12). Especially for the consecutive 2 years of 2023 and 2024, this contagious disease “Baotou” even caused the complete crop cultivation failure of the China’s largest *G. lemaneiformis* farm (Rong Cheng farming region) during the peak cultivation season (12). However, the information of the pathogen that causing the “Baotou” disease remains unclear at present.

Intensive studies have been conducted on the pathogens of various algal rot diseases (13). Alginic acid decomposing bacteria could cause rot disease when *Saccharina japonica* sporeling was incubated in stressful environmental conditions (14). *Pseudoalteromonas, Vibrio,* and *Halomonas* may be the potential pathogenic bacteria associated with the Hole-Rotten Disease of *S. japonica* (15). Interestingly, *V. alginolyticus* was found to be a beneficial bacterium to *S. japonica* and reduces the bleaching disease risk of *S. japonica* (16). *Vibrio* sp. P11 promoted ice-ice disease in *Kappaphycus alvarezii* (17, 18). Syafitri et al. (19) found that the ice-ice disease of *K. alvarezii* was mainly caused by three types of bacteria: *Alteromonas macleodii, Pseudoalteromonas issachenkonii*, and *Aurantimonas coralicida*, among which *A. macleodii* exhibited the strongest pathogenicity. The yellow spot disease (YSD) that occurred in *conchocelis* sporeling cultures of *Pyropia* could be induced by *Vibrio medierranei* 117-T6 (20). Ding and Ma (21) reported that in the red rot diseases of *Porphyra yezoensis*, both *Pythium porphyrae* and *Olpidiopsis* sp. were observed as dominant bacteria. Agar-digesting Vibrio species from the rotten thallus of *Gracilariopsis heteroclada* was isolated and characterized by Lavina-Pitogo (22) and Martinez and Padilla (23). *Thalassospira* sp. and *Vibrio parahaemolyticus*, which could induce the necrosis of healthy tips on *G. lemaneiformis*, were isolated by Sun et al. (11). *Agarivorans albus*, *Aquimarina latercula* (*T*), and *Brachybacterium* sp., which can bleach the healthy *G. lemaneiformis*, were isolated from the bleached *G. lemaneiformis* (10).

Despite the “Baotou” disease continuously and seriously affecting the cultivation of *G. lemaneiformis*, there is still a lack of research on the pathogen. Considering the enormous impact of “Baotou” disease on the production of *G. lemaneiformis*, the pathogen of “Baotou” disease was isolated and identified in this study.

## RESULTS

### Bacterial communities on healthy and rotten algae

**Fig 1:**
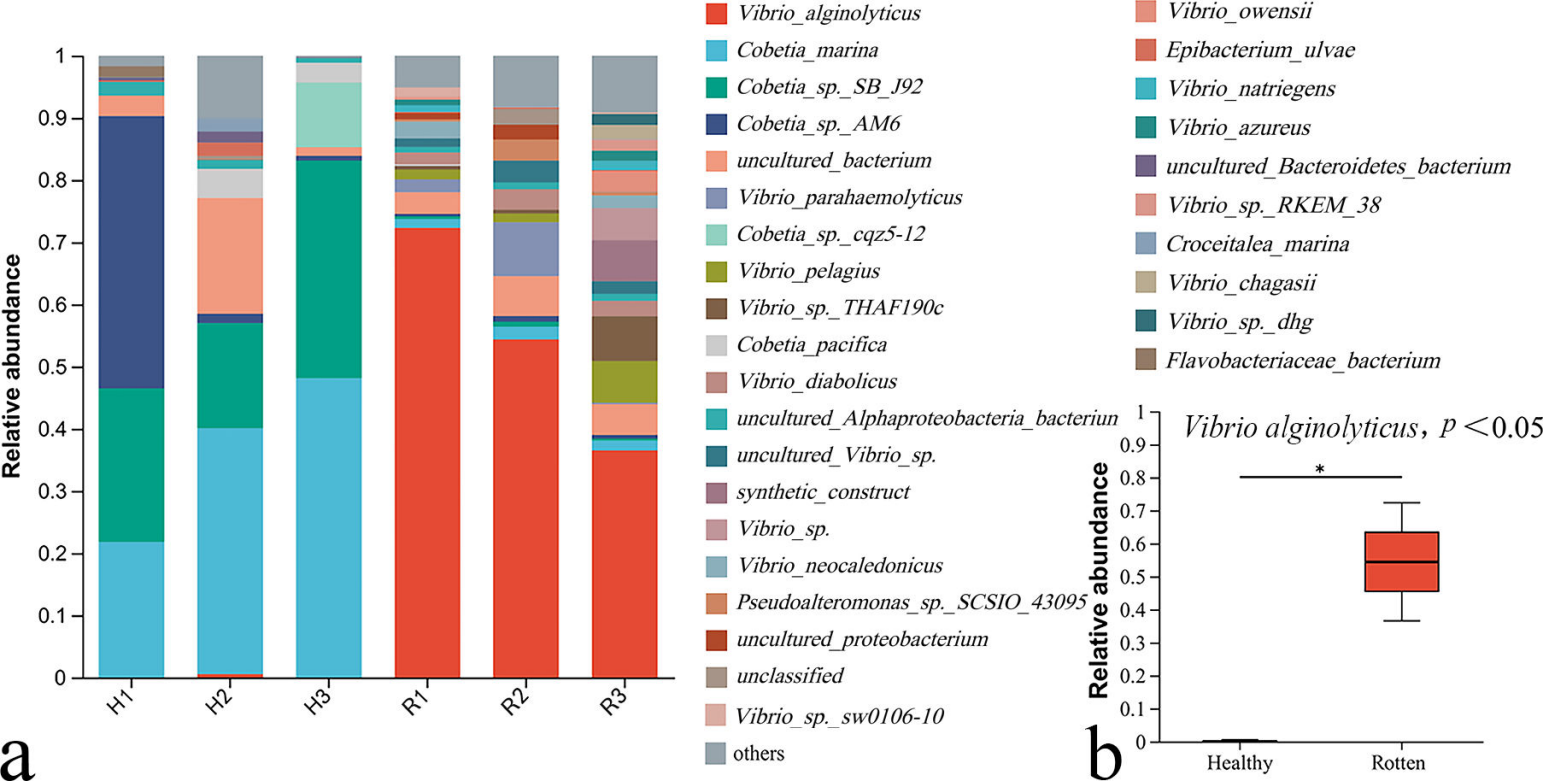
Bacterial communities on healthy and rotten *G. lemaneiformis* thalli. (**a**) The relative abundance of detectable bacteria on healthy (H) and rotten (R) thalli. (**b**) The relative abundance of *V. alginolyticus* on healthy and rotten thalli.

*Vibrio alginolyticus* is the dominant bacterium on the rotten samples (Fig. 1a) and accounted for 72.32%, 54.37%, and 36.53% of the total microbial community on samples R1, R2, and R3, respectively (Fig. 1a). In contrast, *V. alginolyticus* is not detected on the healthy samples of H1 and H3, and only accounted for only 0.56% of the H2 sample. On average, the *V. alginolyticus* accounted 54.41% of the total microbial community on rotten samples, which was significantly higher than that on the heathy samples (0.189%, Fig. 1b).

### Bacterial communities in seawater near healthy and rotten algae

**Fig 2:**
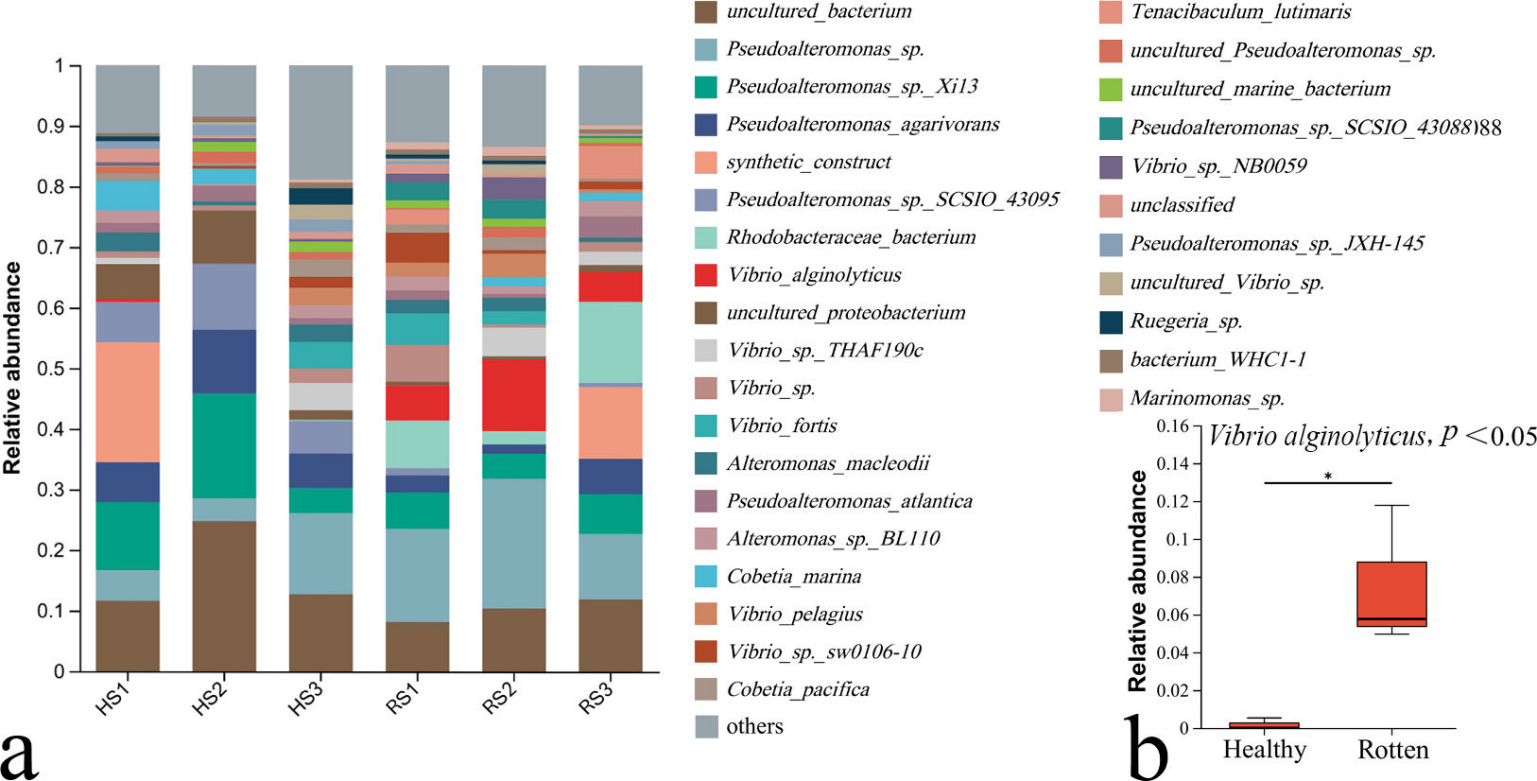
Bacterial communities in seawater near healthy (HS) and rotten (RS) *G. lemaneiformis* thalli. (**a**) The relative abundance of detectable bacteria in the seawater bacterial community near healthy and rotten thalli. (**b**) The relative abundance of *V. alginolyticus* in the seawater near healthy and rotten thalli.

*V. alginolyticus* accounted for 5.58%, 11.78%, and 4.96% of the total microbial community on samples RS1, RS2, and RS3, respectively (Fig. 2a). In contrast, *V. alginolyticus* is not detected on the healthy samples of HS2 and HS3 and only accounted for 0.53% of the HS1 sample. On average, the *V. alginolyticus* accounted 7.5% of the total microbial community in the seawater near rotten samples, which was significantly higher than that in the seawater near heathy samples (0.18%, Fig. 2b).

### Isolated bacteria and their pathogenicity

**Fig 3:**
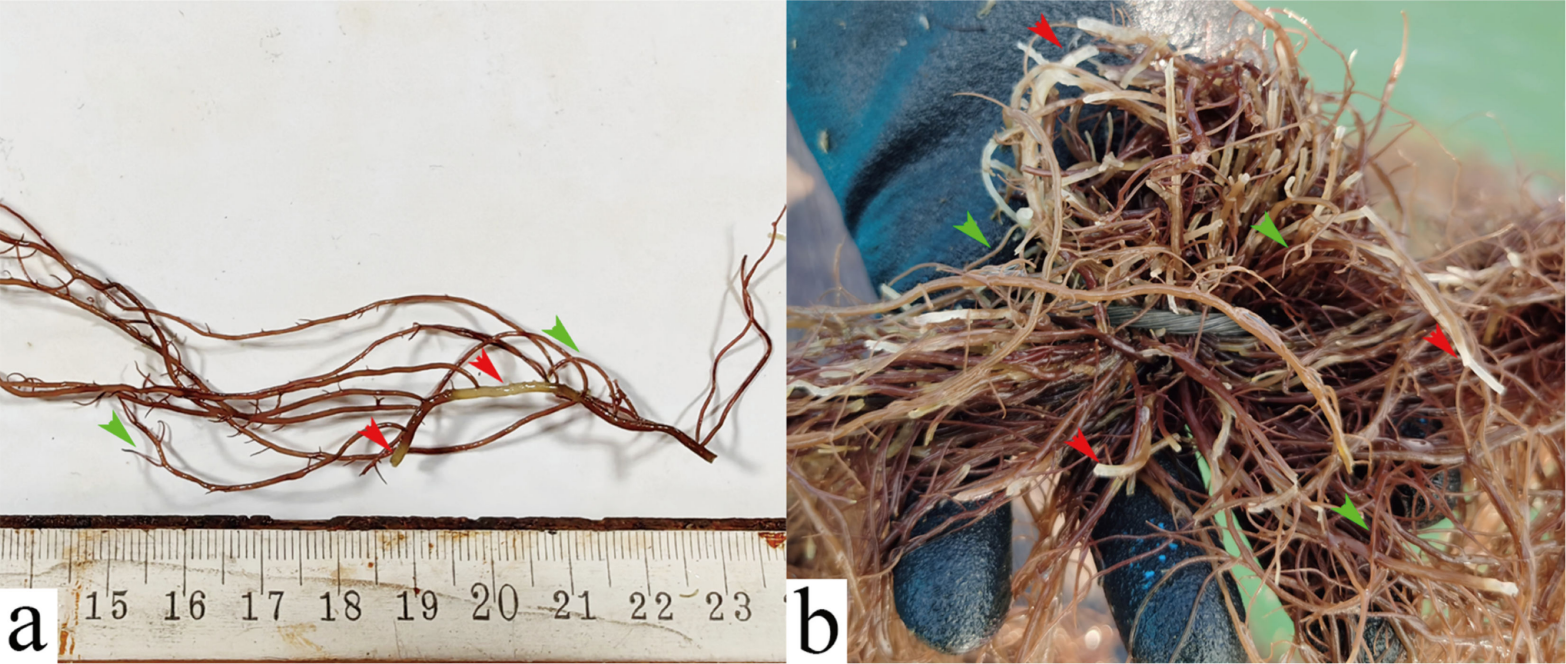
Rotten *G. lemaneiformis* after infection. (**a**) Rotten *G. lemaneiformis* infected by strain ZB10 in the lab. (**b**) Rotten *G. lemaneiformis* with “Baotou” disease in the farm. Red arrows point to the rotten main trunk, and green arrows point to healthy tips.

Forty-nine colonies were obtained for pathogenicity testing, of which 10 strains could induce the rotting of *G. lemaneiformis*. ZB10 exhibited the highest pathogenicity in the 10 strains of bacteria. Six different species of bacteria, which could cause the rotting of *G. lemaneiformis* thalli, were identified by the 16S rRNA phylogenetic analysis (Table 1; supplementary Figs). The pathogenicity of ZB10 and ZB7 was significantly higher than that of the other species at 72 h. The main rotting part caused by “Baotou” disease is the trunk of the algae (Fig. 3a and b). The symptom caused by the ZB10 and ZB7 was the rotting of the main trunk of the algae that was consistent with the “Baotou” disease occurring in farms (Fig. 3a and b).

**TABLE 1:** Isolated pathogenic bacteria and their pathogenicity at 72 h (*n* = 9)

Strain	Specific name	Average rotten number	Significance^*a*^
ZB10	*Vibrio alginolyticus*	2.8	a
ZB7	*Vibrio alginolyticus*	2.4	a
ZB 11	*Pseudomonas putida*	1.9	b
ZB 36	*Pseudomonas putida*	1.8	b
ZB 9	*Shewanella* sp.	1.7	b
ZB47	*Shewanella* sp.	1.7	b
ZB49	*Providencia* sp.	1.7	b
ZB 41	*Providencia rettgeri*	1.6	b
ZB 48	*Providencia rettgeri*	1.6	b
ZB21	*Pseudomonas* sp.	0.9	c
Control	Control	0.3	d

^*a*^ Different letters show significant differences at *P* < 0.05 between different strains.

### Morphology of the pathogenic bacteria

**Fig 4:**
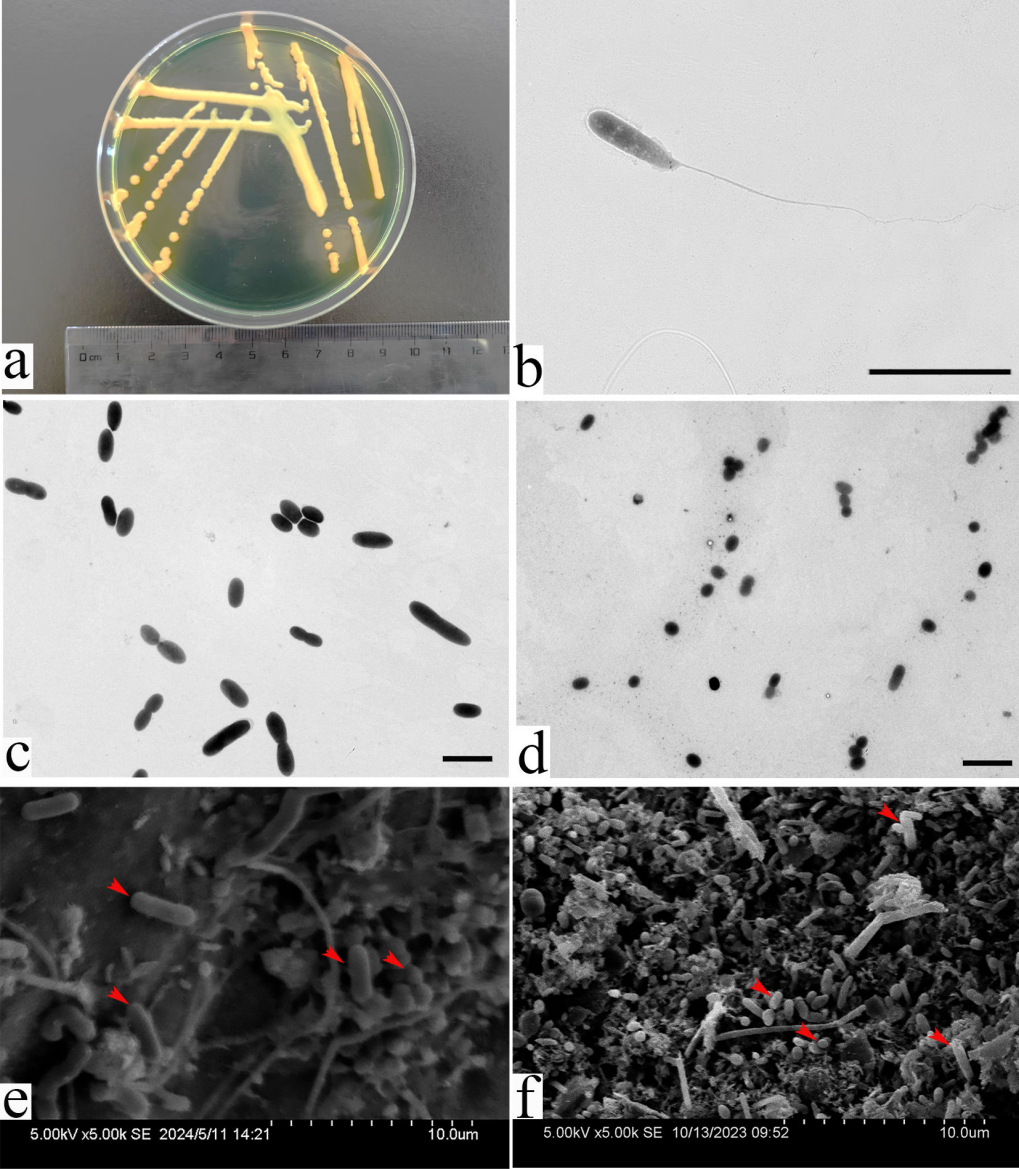
Morphology of the bacteria ZB10. (**a**) Colony morphology of ZB10 on TCBS medium. (**b**) Morphology of the bacteria ZB10 under the TEM with a magnification of 4K. (**c**) Large cells of ZB10 under the TEM with a magnification of 0.7K. (**d**) Small cells of ZB10 under the TEM with a magnification of 0.7K, small ones are only 1 µm long. (**e**) Surface views of the rotten algal thalli derived from lab infection treatment under SEM with a magnification of 5K. (**f**) Surface views of the algal thalli from farm with “Baotou” disease under SEM with a magnification of 5K. Bars: b, c, and d : 5 µm. Red arrows point to pathogenic bacteria *V. alginolyticus*.

The colonies of ZB10 were bright yellow protruding on TCBS medium, with a single-colony diameter of about 2–3 mm and growing diffusely (Fig. 4a). The cell shapes of ZB10 were rod-shaped or spherical (Fig. 4b through d). One curved flagellum was found on the ZB10 under the TEM. The cell sizes of *V. alginolyticus* varied greatly, with large ones reaching 4 µm long and small ones only 1 µm long or even less. (Fig. 4c and d). All the above morphological characteristics were consistent with the morphological characteristics of *V. alginolyticus* reported by Zhao et al. (24). Combined the 16S rRNA phylogenetic analysis and morphological results, ZB10 was identified as *V. alginolyticus*. A large number of *V. alginolyticus* cells were found to be attached to the decaying thalli surface of *G. lemaneiformis* after 72 h of co-culturing and infecting with the *V. alginolyticus* (Fig. 4e). In addition, *V. alginolyticus* can be easily re-isolated from the decayed algal thalli infected in the laboratory. Furthermore, a large number of rod-shaped and spherical bacterial cells similar to those found in the lab infection experiments were also discovered on the *G. lemaneiformis* thalli with “Baotou” disease (Fig. 4f). However, the size of the *V. alginolyticus* found on the “Baotou” disease algae collected from the farm was smaller than that induced in the lab (Fig. 4e and f).

## DISCUSSION

Two strains of pathogenic bacteria, *Thalassospira* sp. and *Vibrio parahaemolyticus*, which could cause the tip rotting of *G. lemaneiformis* was isolated by Sun et al. (11). Pathogenic bacteria, *Agarivorans albus*, *Aquimarina latercula,* and *Brachybacterium* sp., which could induce the bleaching of *G. lemaneiformis*, were isolated by Liu et al. (10). However, the pathogenic bacteria isolated by Sun et al. (11) and Liu et al. (10) were not the dominant bacteria on the rotten *G. lemaneiformis* with “Baotou” disease (Fig. 1a) and were also not found in the seawater of the *G. lemaneiformis* culture area (Fig. 2a). In this study, *V. alginolyticus* was verified to be the dominant bacterium on rotten algae infected by “Baotou” disease (Fig. 1a). In contrast, *V. alginolyticus* was not detected on the healthy samples of H1 and H3 (Fig. 1a). Besides, the *V. alginolyticus* accounted 7.5% of the total microbial community in the seawater near “Baotou” samples, which was significantly higher than that in the heathy samples (0.18%, Fig. 2b). The results of bacterial communities showed that *V. alginolyticus* might have a close relationship with the outbreak of “Baotou” disease.

ZB10 exhibited the highest pathogenicity in the 10 strains of bacteria which could cause the rotting of *G. lemaneiformis*. Moreover, the symptom caused by the ZB10 was the rotting of the main trunk of the algae that was consistent with the “Baotou” disease occurring in farms. The alga with “Baotou” disease has healthy tips, while its trunk is the main rotting part (Fig. 3b), that is different from the characteristics reported by Sun et al. (11). ZB10 was identified as *V. alginolyticus* by morphological and molecular identification. Its morphology was the same as that reported by Zhang et al. (25). The results of the microbial community experiments and the symptoms caused by the *V. alginolyticus* both indicated that *V. alginolyticus* was the pathogen of the “Baotou” disease. Besides, a large number of *V. alginolyticus* cells were found to be attached to the decaying thalli surface of *G. lemaneiformis* after 72 h of co-culturing and infection with the *V. alginolyticus*. In addition, *V. alginolyticus* can be easily re-isolated from the decayed algal thalli infected in the lab. Furthermore, a large number of rod-shaped and spherical bacterial cells identified as *V. alginolyticus* by morphological identification were found to be attached to the “Baotou” disease infected thalli collected from the farm (Fig. 4f). Based on the Koch’s postulates and the above results, the *V. alginolyticus* was identified as the pathogen of “Baotou” disease.

Interestingly, *V. alginolyticus* is the pathogen to “Baotou” diseased *G. lemaneiformis* in this study, but a beneficial bacterium to *S. japonica* (16). *G. lemaneiformis* and *S. japonica* are often co-cultured in China due to their growth periods overlapped for about 3 months. These two species of seaweeds are also often plagued with bleaching diseases during their high-density commercial cultivation in recent years (12, 26). The study seems to remind us that *V. alginolyticus* is closely involved in the competition or co-existence between *G. lemaneiformis* and *S. japonica* co-cultured in China. Besides, *V. alginolyticus* is also pathogen to *Haliotis diversicolor*, *Paleopneustes cristatus, Ruditapes decussatus,* and *Litopenaeus vannamei* (27–30). Interestingly, both *G. lemaneiformis* and *S. japonica* are the main feed for abalone and sea urchin. Considering the serious “Baotou” disease disaster in the cultivated *G. lemaneiformis* and the rich ecological role of *V. alginolyticus*, more research should be devoted to it to further clarify its biological and ecological characteristics.

Collectively, *V. alginolyticus* was the dominant bacterium on *G. lemaneiformis* with “Baotou” disease. In contrast, there were very few *V. alginolyticus* on the healthy samples. *V. alginolyticus* could cause the rotten symptoms of *G. lemaneiformis* in the lab, and the symptoms it caused were consistent with those of “Baotou” disease. Besides, *V. alginolyticus* can be easily re-isolated from the rotten algal thalli infected in the lab. A large number of *V. alginolyticus* cells were found to be attached to the algal thalli with “Baotou” disease and the rotten thalli acquired by the lab *V. alginolyticus* infection treatment. *V. alginolyticus* strictly conforms to the four criteria of Koch’s postulates regarding pathogens and was proven to be the pathogen causing “Baotou” disease in cultivated *G. lemaneiformis* (31, 32).

## MATERIALS AND METHODS

### Source of experimental seaweed

*G. lemaneiformis* used for the experiment was collected from the Ailun Bay farm in Rongcheng City, Shandong Province, China (37°10′ N, 122°34′ E) between June and September 2023. Experiments were carried out immediately after they were collected.

### Collection of the bacterial community samples associated with the *G. lemaneiformis*

The healthy and rotten thalli (5 g) were put in 50 mL tubes, respectively, and then incubated in the liquid nitrogen immediately after they were collected (three replicates for each group). Then, the healthy (H) and rotten (R) thalli were used for studying the bacteria communities attached to the *G. lemaneiformis*. Seawater about 0.5 m away from the healthy and rotten *G. lemaneiformis* thalli was collected, respectively. Then, 1 L of seawater was filtered by 0.2 µm filter membranes, respectively, to obtain bacterial community samples in the seawater around the healthy (HS) and rotten (RS) *G. lemaneiformis*. The filter membranes were put in 5 mL tubes, respectively, and incubated in the liquid nitrogen immediately after the bacteria were collected (three replicates for each group).

### Analysis of the bacterial community structure

Genomic DNA of bacteria was extracted using the Power Soil DNA Isolation Kit (DNeasy) according to manufacturer’s instructions. The 16S rRNA full sequence regions was amplified by PCR. Purified products were pooled in equimolar, and DNA library was constructed using the SMRT bell prep kit 3.0 (Pacifc Biosciences, CA, USA) according to PacBio’s instructions. Purified SMRT bell libraries were sequenced on the Pacbio Sequel IIe System (Pacifc Biosciences, CA, USA) by Majorbio Bio-Pharm Technology Co. Ltd. (Shanghai, China). High-fidelity (HiFi) reads were obtained from the subreads, generated using circular consensus sequencing via SMRT Link v11.0. 485518 sequences were denoised using the DADA2 (33) plugin in Quantitative Insights Into Microbial Ecology (QIIME2 v.2020.2) (34). After denoising, 217,602 sequences (ranging from 10,105 to 27,654 per sample) were obtained, and finally, these sequences were clustered into 1,443 amplicon sequence variants (ASVs). Taxonomic assignment of ASVs was performed using the Vsearch consensus taxonomy classifier implemented in Qiime2 and the SILVA 16S rRNA database (v138). Stacked bar plot conducted by R (v3.3.1) was used to identify the most abundance bacterial communities on species levels, the top 30 species in terms of abundance in the samples were shown.

### Isolation of pathogenic bacteria

The rotten seaweed tissue (0.2 g) was homogenized in 3 mL of sterile seawater for 3 min at room temperature (23°C) and then was centrifuged at 800 × *g* for 5 min. After centrifugation, 0.1 mL of supernatant was diluted by 10, 100, 1,000, and 10,000 times, and 0.1 mL of the different dilutions was spread onto Tryptic Soy Broth (TSB) solid culture medium. After 48 h of cultivation (23°C), single bacterial colony was isolated by TSB plate streaking cultivation, and this process was repeated three times to obtain pure bacterium.

### Pathogenicity assessment

Acquired single bacterial colonies were picked from the solid culture medium and transferred into 50 mL of sterile TSB liquid culture medium. After 24 h of cultivation at 23°C in a shaker (shake rate of 110 rpm), the cultures were centrifuged at 12,000 *g* for 10 min. Sediment was collected and diluted in 500 mL of sterile seawater to obtain a diluted bacterial suspension. Then, healthy algal thalli (weighting 5 g) were incubated in the bacterial suspension (500 mL) for 8 h at 21°C in the dark. After incubation, the seaweed (5 g) was cultivated in different breeding ponds containing 200 L seawater (32 psu, changed every day), at a temperature 21°C, light 60 µmol m^−2^s^−1^ at 14L:10D photoperiod. According to the method of Pang et al. (12), the number of rotten points on each algal thallus was recorded at 0, 24, 48, and 72 h. Two replicates were set for each single bacterial colony, along with two control groups consisting of 500 mL of sterile seawater.

After the first time of preliminary pathogenicity assessment for each bacterial colony, those colonies exhibiting pathogenicity were subjected to further infection validation test. Ten replicates were set for each bacterial colony, along with 10 control groups. The number of rotten points on each algal thallus was recorded at 0, 24, 48, and 72 h according to the method of Pang et al. (12).

### 16S rRNA sequencing and phylogenetic analysis of isolated bacteria

Total DNA was extracted from 1 mL bacterial solution using Ezup Column Bacteria Genomic DNA Purification Kit, following the kit protocol. Then, 16S rRNA was amplified using the method that Gu et al. described (35). Successful PCR products were purified and sequenced by a commercial company (Sangon, China). The Blast algorithm at the National Center for Biotechnology Information (http://www.ncbi.nlm.gov/blast/) was used to compare the nucleotide sequences. Relevant sequences were obtained from GenBank for phylogenetic analysis. A maximum likelihood (ML) phylogenetic tree was constructed with the MEGA 11 program (36). The reliability of the branching was tested by bootstrap re-sampling (1,000 pseudo-replicates).

### Observation of bacterial morphology on seaweed

Rotten seaweeds (three replicates for each type) were collected from the farm and lab pathogenicity assessment experiment, keeping them in their original state, and then proceeded with scanning electron microscopy observation immediately. The processing procedure followed the method described by Pang et al. (12). Then, samples were observed by scanning electron microscopy (Hitachi S-3400).

### Observation of pathogenic bacterial morphology

Transmission electron microscopy (TEM) was used to observe the cell morphology of bacteria. The sample preparation method was modified from Ma et al. (16). Bacterial suspension (OD_600_ = 1.3, 20 mL) was centrifuged at 3,000 × *g* for 10 min and resuspended in 2.5% (wt/vol) glutaraldehyde for 10 min. Then, 10 µL of suspension was moved to a carbon-coated copper grid for 10 min. Finally, the sample was observed by transmission electron microscope (Hitachi-HT7700).

### Statistical analysis

Statistical analyses were performed using IBM PASW Statistics 18. One-way ANOVA and the least significant difference (LSD) at *P* < 0.05 were used to test the significant differences.

## Data Availability

Data are available on request to the corresponding author.

## SUPPLEMENTAL MATERIAL

Fig. S1 to S10 mbio.03120-24-s0001.docx Phylogenetic evolutionary trees of some bacteria in the text.

ASM does not own the copyrights to Supplemental Material that may be linked to, or accessed through, an article. The authors have granted ASM a non-exclusive, world-wide license to publish the Supplemental Material files. Please contact the corresponding author directly for reuse.
