# Supplementary material for: Vibrio alginolyticus is the pathogen of “Baotou” disease causing serious damage to Gracilariopsis lemaneiformis cultivation in China
Source: mBio. 2024 Dec 11;16(1):e03120-24. doi: 10.1128/mbio.03120-24 (PMC11708012; doi:10.1128/mbio.03120-24)
Supplement: Fig. S1 to S10 — Phylogenetic evolutionary trees of some bacteria in the text. [file mbio.03120-24-s0001.docx]

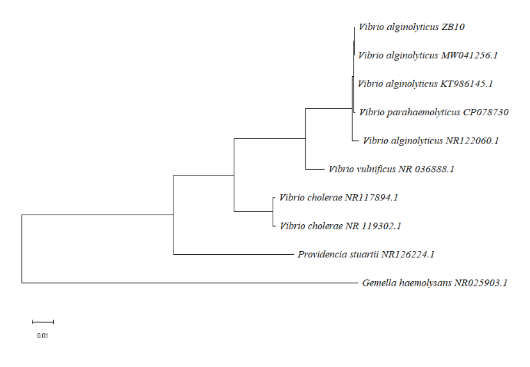


Fig.S1. Neighbour-joining phylogenetic tree of ZB10 based on 16S rRNA sequences. The phylogenetic tree was established by the MEGA 11, with bootstrap replications of 1000. GenBank accession no of ZB10 on NCBI is PQ032495 at [https://www.ncbi.nlm.nih.gov/nuccore/ PQ032495](https://www.ncbi.nlm.nih.gov/nuccore/PQ643767)


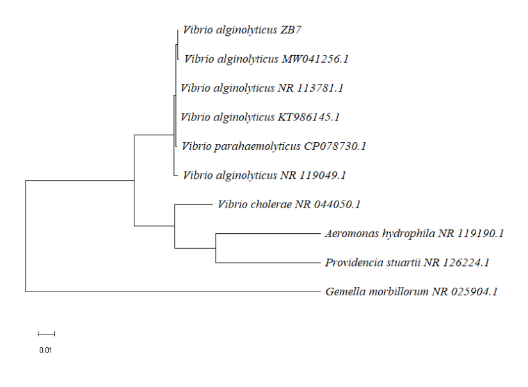


Fig.S2. Neighbour-joining phylogenetic tree of ZB7 based on 16S rRNA sequences. The phylogenetic tree was established by the MEGA 11, with bootstrap replications of 1000. GenBank accession no of ZB7 on NCBI is PQ012459 at <https://www.ncbi.nlm.nih.gov/nuccore/PQ012459>


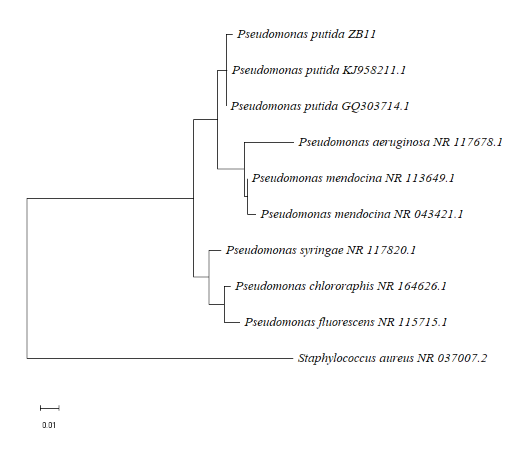


Fig.S3. Neighbour-joining phylogenetic tree of ZB11 based on 16S rRNA sequences. The phylogenetic tree was established by the MEGA 11, with bootstrap replications of 1000. GenBank accession no of ZB11 on NCBI is PQ643767 at <https://www.ncbi.nlm.nih.gov/nuccore/PQ643767>


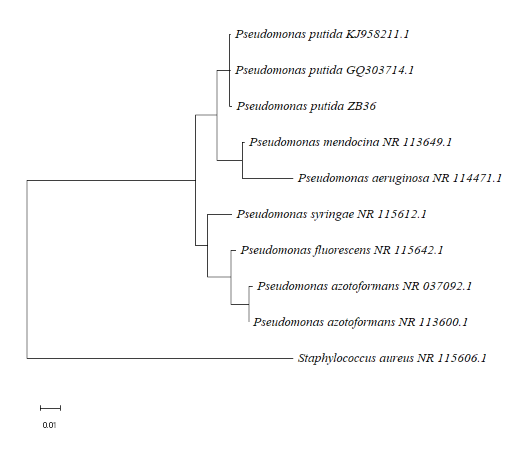


Fig.S4. Neighbour-joining phylogenetic tree of ZB36 based on 16S rRNA sequences. The phylogenetic tree was established by the MEGA 11, with bootstrap replications of 1000. GenBank accession no of ZB36 on NCBI is PQ012461 at <https://www.ncbi.nlm.nih.gov/nuccore/PQ012461>


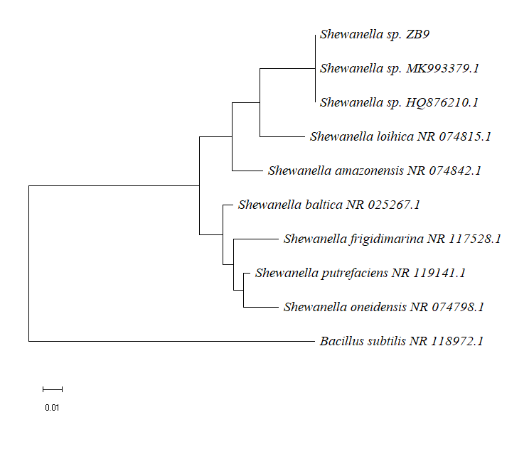


Fig.S5. Neighbour-joining phylogenetic tree of ZB9 based on 16S rRNA sequences. The phylogenetic tree was established by the MEGA 11, with bootstrap replications of 1000. GenBank accession no of ZB9 on NCBI is PQ012460 at <https://www.ncbi.nlm.nih.gov/nuccore/PQ012460>


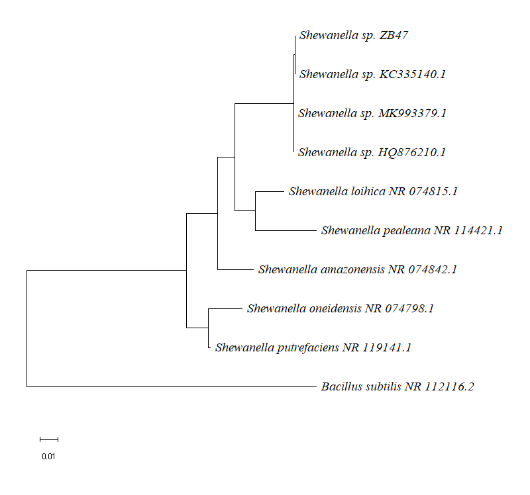


Fig.S6. Neighbour-joining phylogenetic tree of ZB47 based on 16S rRNA sequences. The phylogenetic tree was established by the MEGA 11, with bootstrap replications of 1000. GenBank accession no of ZB47 on NCBI is PQ032496 at <https://www.ncbi.nlm.nih.gov/nuccore/PQ>032496


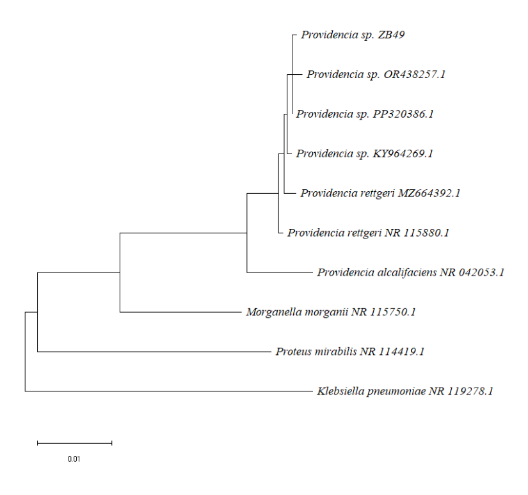


Fig.S7. Neighbour-joining phylogenetic tree of ZB49 based on 16S rRNA sequences. The phylogenetic tree was established by the MEGA 11, with bootstrap replications of 1000. GenBank accession no of ZB49 on NCBI is PQ032497 at <https://www.ncbi.nlm.nih.gov/nuccore/PQ032497>


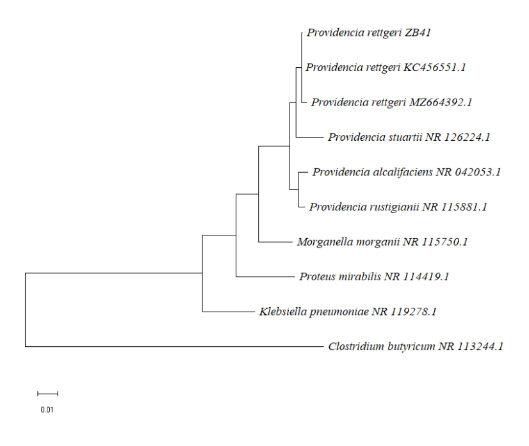


Fig.S8. Neighbour-joining phylogenetic tree of ZB41 based on 16S rRNA sequences. The phylogenetic tree was established by the MEGA 11, with bootstrap replications of 1000. GenBank accession no of ZB41 on NCBI is PQ012463 at <https://www.ncbi.nlm.nih.gov/nuccore/PQ012463>


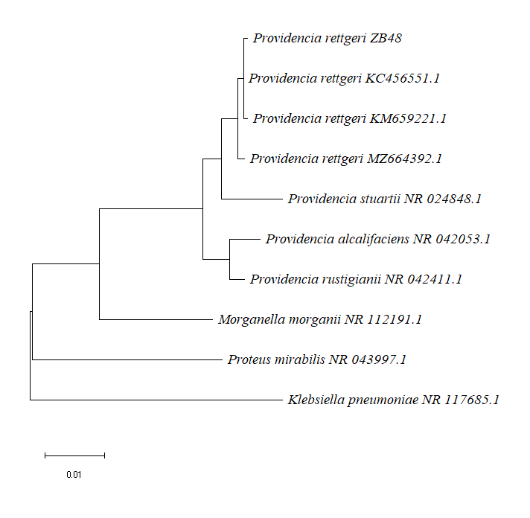


Fig.S9. Neighbour-joining phylogenetic tree of ZB48 based on 16S rRNA sequences. The phylogenetic tree was established by the MEGA 11, with bootstrap replications of 1000. GenBank accession no of ZB48 on NCBI is PQ012464 at <https://www.ncbi.nlm.nih.gov/nuccore/PQ012464>


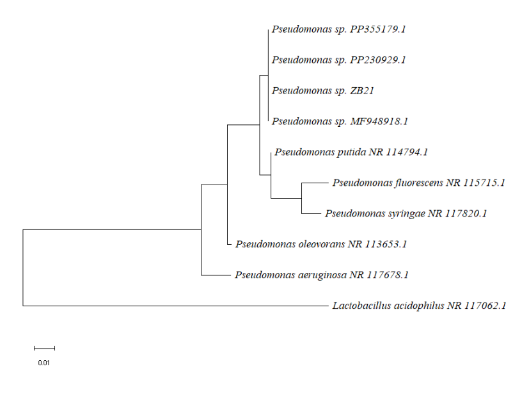


Fig.S10. Neighbour-joining phylogenetic tree of ZB21 based on 16S rRNA sequences. The phylogenetic tree was established by the MEGA 11, with bootstrap replications of 1000. GenBank accession no of ZB21 on NCBI is PP917733 at <https://www.ncbi.nlm.nih.gov/nuccore/PP917733>
